# The association between family members’ migration and cognitive function among people left behind in China

**DOI:** 10.1371/journal.pone.0222867

**Published:** 2019-09-26

**Authors:** Yosuke Inoue, Annie Green Howard, Bo Qin, Aki Yazawa, Andrew Stickley, Penny Gordon-Larsen

**Affiliations:** 1 Carolina Population Center, The University of North Carolina at Chapel Hill, Chapel Hill, North Carolina, United States of America; 2 Department of Biostatistics, Gillings School of Global Public Health, The University of North Carolina at Chapel Hill, Chapel Hill, North Carolina, United States of America; 3 Department of Population Science, Rutgers Cancer Institute of New Jersey, New Brunswick, New Jersey, United States of America; 4 Department of Social and Behavioral Sciences, Harvard T. H. Chan School of Public Health, Harvard University, Boston, Massachusetts, United States of America; 5 The Stockholm Center for Health and Social Change (SCOHOST), Södertörn University, Huddinge, Sweden; 6 Department of Nutrition, Gillings School of Global Public Health, The University of North Carolina at Chapel Hill, Chapel Hill, North Carolina, United States of America; Appalachian State University, UNITED STATES

## Abstract

While internal migration is widely occurring in countries across the world and older people are more likely to be left behind by family members who out-migrated to other locations, little attention has been paid to the cognitive health of those people who have been left behind (PLB). Understanding how these demographic patterns relate to older persons’ cognitive health may inform efforts to reduce the disease burden due to cognitive decline. Data came from the China Health and Nutrition Survey in 1997, 2000 and 2004. Participants aged 55 to 93 who participated in a cognitive function screening test (score range: 0–31) in two or more waves and provided information on family members’ migration (n = 1,267) were included in the analysis. A mixed linear model was used to investigate the association between being left behind by any members who had not resided in the household for at least 6 months at baseline and cognitive function. Approximately 10% of the participants had been left behind by family members who migrated out of their communities. A significant interaction was observed in relation to cognitive function between being left behind and the number of years from the first test. Specifically, there was a less steep decline in cognitive function of PLB compared to people not left behind. This longitudinal study showed that PLB tended to have a higher cognitive function compared to those not left behind due to their relatively stable transition in cognitive function during the study period.

## Introduction

Given that internal migration is now widely occurring in countries across the world, older people are more likely to be left behind by their adult children who leave for other locations to engage in migratory work [1]. Accordingly, a growing body of literature has paid attention to the health of people left behind (PLB) by migrant family members [2–4], but has had different hypotheses and produced conflicting results.

For example, some studies have assumed that the outmigration of family members might result in negative health consequences as it may lead to diminished social support and reduced household labor [5]. Support for this supposition has been found in a study by Lu et al. [6] who showed that in China, individuals living in households with a migrant family member were more likely to have depressive symptoms, which is itself a risk factor for dementia and cognitive decline, than those in households without migrants. On the other hand, other researchers have argued that the migration of family members might bring benefits such as increased remittance, which may result in better health outcomes. For example, Abas et al. [7] found that in Thailand, parents with children who out-migrated had a lower risk of depression compared to parents without migrating children.

Another line of research on the health of migrants’ family members has focused on the ‘empty-nest elderly’, particularly those living in rural China, where large-scale rural-to-urban migration has been occurring [8,9] and an increase in the number of such people has become a matter of public concern [10]. This term refers to older parents living alone or only with a spouse and it is of note that the empty-nest elderly and PLB have much in common but are different in that the latter doesn’t necessarily live alone or with only a spouse. Several studies have reported that the empty-nest elderly tended to have a worse physical and psychological health condition compared to those who were not empty-nest elderly [11–15], albeit with some exceptions [15,16].

The current study extended the previous research in several ways. First, as yet, little research on PLB has examined the association between being left behind and cognitive function, which declines progressively with aging [17]. While doing so, we accounted for living arrangements (e.g. living alone) to disentangle the effect of being left behind and that of being empty-nest elderly, which has been previously shown to be associated with a lower cognitive function [18]. Understanding how being left behind by family members who out-migrated to other locations relates to older persons’ cognitive health may inform efforts to reduce the disease burden due to cognitive decline in this population. Second, we used a longitudinal study design whereas most of the previous research on this topic (excepting only a few studies [19,20]) has used cross-sectional data [4,6,21,22]. Our use of longitudinal data allows for the sequence of migration prior to cognitive decline since parental cognitive status might influence family member migration (i.e., selective migration) [23]. Third, we focused on China, a country where large-scale rural-to-urban migration has been occurring [8]. Findings from this study may facilitate our understanding of the association between being left behind and cognitive function in other developing countries.

In short, the aim of this study was to investigate the association between family out-migration and the cognitive function of older persons who had been left behind by family members who had not lived in the household for at least 6 months at baseline, using information collected in the China Health and Nutrition Survey (CHNS).

## Materials and methods

### Setting

We used data from three waves of the CHNS, which is a longitudinal household-based survey with ongoing data collection in 228 communities in nine provinces of China (i.e., Heilongjiang, Liaoning, Shandong, Henan, Jiangsu, Hunan, Hubei, Guangxi, and Guizhou) [24]. Using a multistage, random cluster design, a stratified probability sample was used to select counties and cities stratified by income using State Statistical Office definitions [25]; we selected participating communities and households from these strata. Questionnaires were used to collect demographic, socioeconomic, behavioral, and health information from each household member. The CHNS cohort initially mirrored national age-sex-education profiles [26,27] and the provinces in the CHNS sample contained 44% of China’s population in 2009. A detailed description of the survey procedures has been provided elsewhere [24,28].

### Participants

Cognitive tests were administered among CHNS participants aged 55 years or above in the 1997, 2000 and 2004 waves of the survey by interviewers. For this study, we included adults aged 55 and above at entry who completed the test on at least two separate occasions (age range: 55–93 years old; the participation rate for the baseline cognitive test among eligible CHNS participants was 73%). A total of 1,267 individuals with 2,944 observations were included in the subsequent analysis.

The study met the standards for the ethical treatment of participants and was approved by the Institutional Review Boards of the University of North Carolina at Chapel Hill (07–1963), the China-Japan Friendship Hospital, the Ministry of Health of China, and the Institute of Nutrition and Health, Chinese Center for Disease Control and Prevention.

### Outcome variable

The cognitive screening items in the CHNS included a subset of the items from the Telephone Interview for Cognitive Status–modified [29], which has been previously used in the United States [30] and China [31–33]. The cognitive screening included immediate and delayed recall of a 10-word list (score ranging 0–10 points), counting backward from 20 (0–2 points), serial 7 subtractions from 100 (0–5 points), and orientation (0–4 points). Counting backward and serial 7 subtractions were used to evaluate attention and calculation ability. Orientation was assessed by asking the respondent the current date (1 point each for year, month, and date) and to name the instrument usually used to cut paper (1 point). We summed the scores of all the test items, which gave a total score that ranged from 0 to 31 points, with higher scores indicating better cognitive function.

### Explanatory variables

In accordance with the governmental definition [9], we defined PLB as those individuals who lived in a household where at least one family member had been living outside the study community for six months or longer at entry. While we did not collect detailed information on family members’ migration, it was previously reported that approximately half of China’s population who reside in a location different from their place of household registration migrated across the provincial border (53.9% in 2000 and 50.3% in 2010). In addition, more than 70% of inter-provincial migrants moved for manual labor or business while less than half of intra-provincial migrants migrated for manual jobs (48.0% in 2000 and 37.5% in 2010) [9]. Demographic and socioeconomic covariates included age, sex, educational attainment (illiterate; primary school graduate; junior high school or higher), and annual household income per capita (inflated to 2011, < / ≥ 5000 yuan, equivalent to 773 US dollars as of July 1, 2011). Information on the degree of urbanization as measured by the urbanization index (a CHNS-specific multicomponent continuous scale) [34], living arrangement (living alone; with another member; with two or more members) [18], stability/change in self-rated health (excellent, good, fair and poor) and instrumental activities of daily living (IADL) was also included. Information on self-reported health obtained from the first and last participation in the survey was used to categorize participants into the following categories: “persistently good self-rated health”, “improving self-rated health”, “worsening self-rated health”, and “persistently poor self-rated health”. People were categorized as having IADL difficulty if they reported having difficulty doing any (≥ 1) of the following activities: shopping; cooking; using transportation; managing money; and using a telephone; we then divided respondents’ scores into the following categories: “remained non-disabled”, “improving”, “worsening” and “persistently disabled”. All covariates except for the IADL and self-rated health scores were obtained at the time of the first cognitive measurement. If participants had missing information for any of the covariates, they were assigned to a ‘missing’ category and included in the analysis.

### Statistical analysis

The characteristics of participants by PLB status were compared using t-tests for continuous variables and Fisher’s exact tests/chi-square tests for categorical variables. A linear mixed-effects model was used to assess the association between being left behind and cognitive function over time. Both the intercept and slope were fitted with random-effects components to account for inter-individual differences in baseline cognitive function and the rate of change [31].

Model 1 included PLB status, age, gender, educational attainment, household income, living arrangement, the urbanization index and an interaction term of these variables with time (the number of years from entry). Model 2 was further adjusted for stability/change in self-rated health and IADL difficulties. We calculated predicted values for the combination of PLB status and study year while holding the covariates at their mean values.

We also conducted sensitivity analyses to examine the robustness of our study findings, where the analysis was restricted to (1) those who did not have poor self-reported health at baseline and (2) those who did not have any IADL difficulties at baseline.

Statistical significance was set at p < 0.05 for the main effects and p < 0.10 for interaction effects (two-tailed). All the analyses were conducted using Stata SE (version 14.0, Stata Corp, College Station, TX).

## Results

### Characteristics of the study participants

The basic characteristics of the study participants, who were followed up for an average period of 5.2 years, are presented in Table 1. There were 857 and 410 people who respectively participated in two and three waves of the survey. At study entry, PLB comprised 9.6% of the study participants. They tended to be younger, to report poor health, and to live in less urbanized communities than those who were not left behind. There was no substantial difference between PLB and people not left behind in relation to sex, education, household income, living arrangement, cognitive function and IADL.

**Table 1:** Basic characteristics of the study participants in China, 1997–2004.

	People left behind (n = 122)		People not left behind (n = 1145)		p-value^a^
Baseline information					
Age, mean [SD]	61.6	[5.9]	64.0	[6.3]	< 0.001
Female, n (%)	60	(49.2)	530	(46.3)	0.543
Education, n (%)					
Illiterate	67	(54.9)	563	(49.2)	0.138
Primary school	26	(21.3)	249	(21.8)	
Junior high school or above	21	(17.2)	287	(25.1)	
Missing	8	(6.6)	46	(4.0)	
Household income, n (%)					
< 5000 yuan	81	(66.4)	717	(62.6)	0.334
≥ 5000 yuan	41	(33.6)	407	(35.6)	
Missing	0	(0.0)	21	(1.8)	
Living arrangement, n (%)					
Living alone	4	(3.3)	48	(4.2)	0.154
Living with another member	27	(22.1)	344	(30.0)	
Living with two or more	91	(74.6)	753	(65.8)	
Urbanization index, mean [SD)	50.2	[17.6]	61.9	[17.7]	< 0.001
Cognitive score, mean [SD]	18.9	[6.3]	19.4	[5.7]	0.398
Time-varying information					
Transition in self-rated health, n (%) ^b^					
Persistently good self-rated health	16	(13.1)	301	(26.3)	0.017
Improving self-rated health	21	(17.2)	175	(15.3)	
Worsening self-rated health	40	(32.8)	322	(28.1)	
Persistently poor self-rated health	44	(36.1)	335	(29.3)	
Missing	1	(0.8)	12	(1.1)	
Transition in instrumental activities of daily living, n (%) ^b^					
Remained non-disabled	77	(63.1)	731	(63.8)	0.859
Improving	15	(12.3)	113	(9.9)	
Worsening	13	(10.7)	149	(13.0)	
Persistently disabled	8	(6.6)	75	(6.6)	
Missing	9	(7.4)	77	(6.7)	

^a^ Participants’ characteristics were compared using t-tests for continuous variables, Fisher’s exact test (education, household income, living arrangement, self-rated health, and instrumental activities of daily living [IADL]), and chi-squares tests (sex) for categorical variables.

^b^ Information on self-rated health and IADL difficulty was obtained at the time of the first and last participation in the survey to determine the degree of stability/change in the scores on these variables across time.

**Fig 1:**
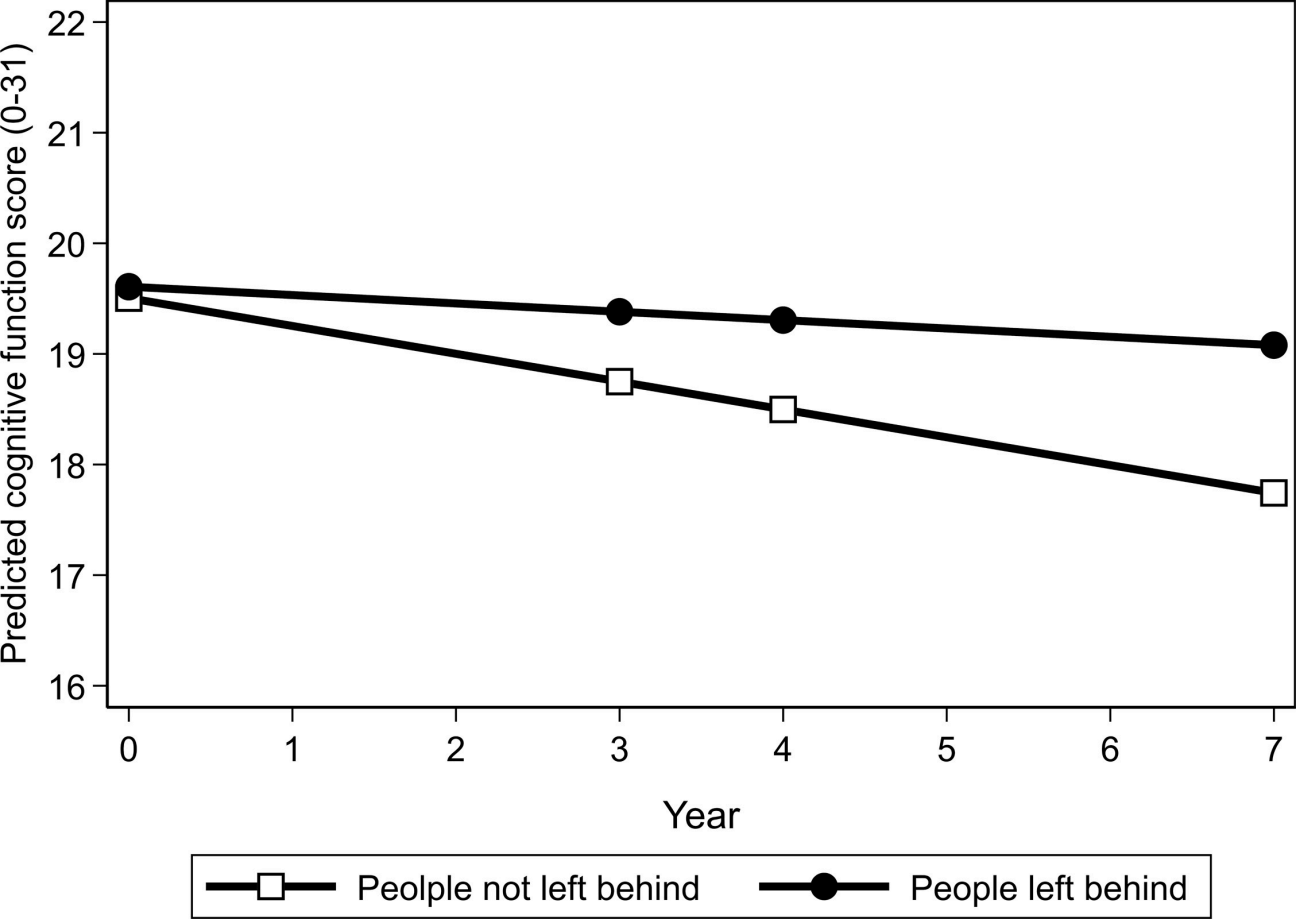
Linear trajectories of cognitive scores among the older Chinese population comparing people left behind and people not left behind by family members who out-migrated to other locations.

The results of a mixed linear regression model investigating the association between being left behind and cognitive function are presented in Table 2. Cognitive function at baseline did not differ either by PLB status or living arrangement (Table 2). The urbanization index was positively associated with cognitive function (coef. = 0.40, 95% CI = 0.24–0.57) (Model 1). The interaction term between being left behind and the number of years from study entry was statistically significant (coef. = 0.20, 95%CI = -0.03, 0.42, p = 0.087). These overall trends did not change after adjusting for across-time variability in IADL difficulty and self-rated health scores while the interaction term became non-significant (p = 0.117) (Model 2); Fig 1 illustrates that the cognitive function score of PLB was much more stable across time compared to that of individuals not left behind which meant that by the end of the study period, cognitive function was higher in PLB.

**Table 2:** Mixed linear model investigating the association between being left behind and cognitive function in China, 1997–2004.

	Model 1		Model 2	
	*coef*.	*95% CI*	*coef*.	*95% CI*
People left behind	-0.07	-0.99, 0.86	0.10	-0.79, 1.00
Living arrangement (ref. living with two or more members)				
Living alone	0.21	-1.17, 1.59	0.15	-1.18, 1.49
Living with another member	-0.07	-0.68, 0.53	-0.13	-0.73, 0.46
Urbanization index	0.40***	0.24, 0.57	0.31***	0.14, 0.48
Year	0.13	-0.60, 0.86	0.02	-0.73, 0.77
Interaction with year				
People left behind × Year	0.20^†^	-0.03, 0.42	0.18	-0.04, 0.40
Living alone × Year	-0.30	-0.67, 0.06	-0.32^†^	-0.67, 0.04
Living with another member × Year	0.07	-0.08, 0.22	0.06	-0.09, 0.20
Urbanization index × Year	0.01	-0.03, 0.06	0.02	-0.03, 0.06

†: p < 0.10

*: p < 0.05

**: p < 0.01

***: p < 0.001

Model 1 was adjusted for age, sex, education, household income, living arrangement and the interaction between these variables and the number of years from study entry. Model 2 was further adjusted for across-time stability/change in self-reported health and instrumental activities of daily living (IADL) difficulties scores.

The interaction term between living alone and the number of years from study entry was inverse (coef. = -0.30, 95%CI = -0.67, 0.06 in Model 1), which suggests that compared to those living with two or more household members, those living alone had experienced a steeper decline in cognitive function during the study period. This observed association became statistically significant when we adjusted for across-time variability in IADL difficulty and self-rated health scores in Model 2.

Sensitivity analyses also showed similar patterns in relation to cognitive function among those who did not have poor self-reported health at baseline (S1 Table and S1 Fig). More specifically, the cognitive function score of PLB was more stable across time compared to that of individuals not left behind. When we confined our analysis to those with no IADL difficulties at baseline, there was no interaction between PLB and the number of years from the baseline, which indicates that there was no difference in the transition in cognitive function by PLB status among old people without any functional limitation.

## Discussion

### Summary of the findings

We used longitudinal data to investigate the association between being left behind as a result of out-migration of family members and cognitive function in Chinese participants aged 55 and above. We found that over the 7-year follow-up period the cognitive function of participants with family members who out-migrated for at least 6 months at baseline remained relatively stable, whereas the cognitive function of individuals who did not have out-migrating family members declined over time. We also found that compared to those living with two or more household members, those living alone at baseline had experienced a steeper decline in cognitive function over the study period.

### Cognitive function of people left behind

The positive benefits associated with family migration might have outweighed the potentially negative effects of migration [6]. Specifically, due to an almost imperceptible level of decline, the cognitive function scores of participants left behind by out-migrating family members became higher than the scores of participants with no out-migrating family members. Although it is uncertain what underlies this comparative stability in cognitive function, different mechanisms might be important. For example, it is possible that being left behind might be associated with an improvement in living standards as a result of the extra income provided by out-migrating family members. This is in line with the result of a study by Du et al. [35] in China showing that having a household member who out-migrated increased the household's income by 8.5–13.1% and by De Brauw et al. [36], who also found that in China out-migration was associated with a 20% increase in investment in housing and other consumer durables. Given that previous research has linked lower income to poorer cognitive function [37,38], this might have explained the comparative stability in cognitive function among those left behind. Alternatively, the reduction in household labor associated with family members’ outmigration may result in a situation where more household responsibility falls on those left behind; Chang et al. [39] showed for instance, that the out-migration of household members in China increased the time spent on farm work and domestic work among those who remained in their original communities. In addition, it has also been widely reported that older generations in rural communities in China are increasingly taking care of grandchildren left behind by out-migrating parents [40]. These increased duties and new responsibilities might be associated with physical and mental health benefits and result in better cognitive functioning. Support for this supposition comes from earlier research which showed that physical activity helps prevent cognitive decline in old people [41,42].

### Living arrangement and cognitive function

Our finding that those living alone at baseline had experienced a steeper decline in cognitive function compared to those living with two or more household members is in line with previous studies that specifically investigated the association between being empty-nest elderly and health [15,43]. In particular, it accords with Duan et al. [18] reporting that the reduction in cognitive function as assessed by the Mini-Mental State Examination and Montreal Cognitive Assessment was larger in the empty-nest elderly living alone and empty-nest elderly living with a spouse than in the control group. They also reported an association between psychological distress associated with being empty-nest elderly and the progression of white matter hyperintensities, suggesting biological mechanisms linking empty-nest elderly status and cognitive decline. Several other studies have reported that the empty-nest elderly suffer from depressive symptoms [44], loneliness [45], and low life satisfaction [46]. Together with these studies, the results of our study suggest that priority should be given to those living alone in the provision of public health services while it seems that being left behind itself does not necessarily adversely affect health.

### Urbanization and cognitive function

We also found that urbanization had an independent effect on the cognitive function of older individuals, with increased urbanization being linked to better cognitive functioning. This is in line with the findings of Jia et al. [47] who showed that urban residents had higher cognitive functioning compared to those in rural communities. While the causes of this seeming urban advantage have yet to be elucidated, there are various factors that have been previously shown to be associated with cognitive function that differ between urban and rural populations in China that might be important in this context. For example, fish consumption, which has been previously linked to cognitive function [31], is approximately twice as high among urban compared to rural Chinese [48]. Similarly, smoking which has also been associated with reduced cognitive function [49] is higher in rural than urban Chinese men [50].

### Limitations

This study has several limitations. First, the cognitive tests in the CHNS were not clinically conducted (e.g., magnetic resonance imaging or positron emission tomography) which might have resulted in misclassification of some subjects in relation to cognitive function. Second, we were missing information on some variables which might have helped us to better understand the association between migratory status and cognitive function such as family debt, the detailed reason for migration, the migration destination, the amount of remittance sent to the household, and on buffering mechanisms against psychological stress due to family separation in rural areas (e.g., bonding social capital [51]). Third, while we used baseline PLB status to avoid reverse causation (i.e., those who had cognitive decline were more likely to be taken care of by family members and less likely to be left behind), PLB status can change over the study period. Fourth, we used information collected in 1997 to 2004; however, our longitudinal findings for China from the initial stage of economic development rather than from the current time can provide an insight on the association between being left behind and cognitive function in other developing countries where rapid economic growth is expected.

## Supporting information

S1 Table Mixed linear model investigating the association between being left behind and cognitive function in China (1997–2004), restricting analyses to those who did not have health problems at baseline. (PDF)

S1 Fig Linear trajectories of cognitive scores among older Chinese adults who did not have health problems at baseline comparing people left behind and people not left behind by family members who out-migrated to other locations. (PDF)

## Abbreviations

CHNS: China Health and Nutrition Survey
IADL: instrumental activities of daily living
PLB: people left behind
