# Supplementary material for: The association between family members’ migration and cognitive function among people left behind in China
Source: PLoS One. 2019 Sep 26;14(9):e0222867. doi: 10.1371/journal.pone.0222867 (PMC6762087; doi:10.1371/journal.pone.0222867)
Supplement: S1 Table — (PDF) [file pone.0222867.s001.pdf]

S1 Table. Mixed linear model investigating the association between being left behind and cognitive function in China (1997 – 2004), restricting analyses to those who did not have health problems at baseline.

	**Self-rated health not poor at baseline (n = 1166)**		**No IADL difficulties at baseline (n = 961)**	
	coef.	*95% CI*	*coef.*	*95% CI*
People left behind	0.32	-0.65, 1.30	0.46	-0.56, 1.49
Living arrangement (ref. living with two or more members)				
Living alone	-0.08	-1.50, 1.33	-0.11	-1.72, 1.49
Living with another member	-0.12	-0.74, 0.51	-0.20	-0.84, 0.45
Urbanization index	0.43***	0.25, 0.60	0.43***	0.24, 0.61
Year	-0.03	-0.79, 0.73	0.37	-0.50, 1.24
Interaction with year				
People left behind × Year	0.22†	-0.02, 0.46	0.12	-0.15, 0.38
Living alone × Year	-0.25	-0.63, 0.12	-0.33	-0.78, 0.12
Living with another member × Year	0.07	-0.09, 0.22	0.05	-0.11, 0.22
Urbanization index × Year	0.02	-0.03, 0.06	0.02	-0.03, 0.07

Models were adjusted for age, sex, education, household income, living arrangement and the interaction between these variables and the number of years from study entry.
†: $p < 0.10$; ***: $p < 0.001$
