# Supplementary material for: The association between family members’ migration and cognitive function among people left behind in China
Source: PLoS One. 2019 Sep 26;14(9):e0222867. doi: 10.1371/journal.pone.0222867 (PMC6762087; doi:10.1371/journal.pone.0222867)

S1 Fig. Linear trajectories of cognitive scores among older Chinese adults who did not have health problems at baseline comparing people left behind and people not left behind by family members who out-migrated to other locations.

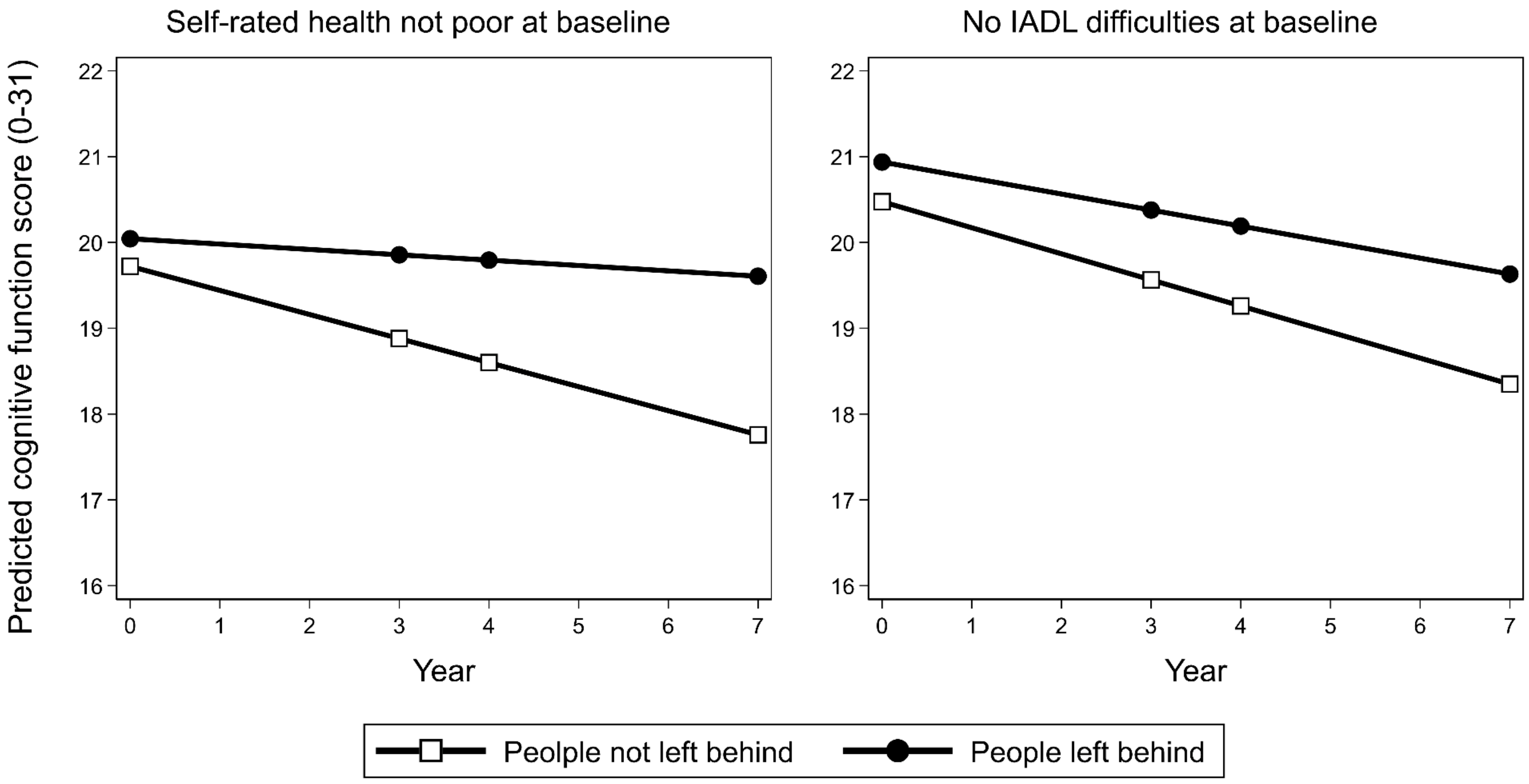

Supplement: S1 Fig — (PDF) [file pone.0222867.s002.pdf]
